# Tree diversity in a tropical agricultural-forest mosaic landscape in Honduras

**DOI:** 10.1038/s41598-022-21280-7

**Published:** 2022-11-03

**Authors:** Marie Ange Ngo Bieng, Diego Delgado-Rodríguez, Sergio Vilchez-Mendoza, Arlene López-Sampson, Edwin García, Norvin Sepúlveda, Eduardo Somarriba

**Affiliations:** 1 CATIE - Centro Agronómico Tropical de Investigación y Enseñanza, 30501 Turrialba, Costa Rica; 2 CIRAD, Université de Montpellier, UR Forêts & Sociétés, 34398 Montpellier, France

**Keywords:** Ecology, Environmental sciences, Agroecology, Ecosystem services

## Abstract

Biodiversity decline in the tropics requires the implementation of comprehensive landscape management where agricultural systems are necessarily an integral element of biodiversity conservation. This study evaluates the potential for taxonomic biodiversity conservation within an intensive livestock-agricultural-forest mosaic landscape in Catacamas, Honduras. Tree sampling was performed in 448 plots set up within different forest and agricultural land uses: secondary forests, agroforestry coffee plantations, agriculture, pastures, live fences and riparian forest. All trees with a minimum diameter at breast height of 10 cm were identified and measured. We characterized their tree structure and diversity, and compared tree diversity between the different uses. The results indicate a high degree of tree species diversity: 375 species identified, belonging to 74 families among the 15,096 trees inventoried across 84.2 hectares, including many rare species (40% of the species registered three individuals or fewer). Biodiversity indices for agroforestry coffee were found equivalent to those for natural secondary forests in the Catacamas landscape. Combining biodiversity conservation and agricultural production is possible in human-pressured tropical landscapes through tree cover maintenance. Enrichment practices combining local producers and technical knowledge may improve tree diversity in agricultural landscapes by prioritizing a mix of forest and introduced tree species (rare and with multiple uses).

## Introduction

Tropical forests, irreplaceable for their role in supporting terrestrial biodiversity conservation^1,2^, are extremely vulnerable to destruction and degradation. Since 1990, most of the deforestation has occurred in tropical regions^3^. Deforestation and degradation of tropical forest areas, and conversion of forest landscapes into agricultural landscapes, is correlated with the worldwide decline in biodiversity in forest landscapes^2^. Many tropical landscapes are subjected to intense human activity, with agricultural land uses inserted in highly fragmented forests.

For example, in Central America, where this study took place, it is estimated that 80% of the region’s original vegetation had become agriculture by the beginning of the twenty-first century^4^. A land use change study over the period 1961–2001 found that the region experienced an average annual rate of reduction in forest cover of 1.2%, with almost half of the original coverage eliminated^5^. As in many tropical countries, deforestation in Central America has primarily been associated with subsistence agriculture and livestock systems^6^. Central America is, however, key for tropical and neotropical biodiversity worldwide. It is a narrow strip land of over half a million km^2^ between South and North America and is considered as one of the planet’s biodiversity hotspots^7^, as the region contains around 7% of the world’s biodiversity. The huge diversity of this region is related to its diversity of forest ecosystems: tropical moist broadleaf forests, tropical dry broadleaf forests, tropical and subtropical coniferous forests, and the decline in its biodiversity is related to the decrease in its forest areas and the increase of its agricultural areas^8^.

Biodiversity decline related to the continuous decrease in forest areas requires the implementation of comprehensive landscape management, involving conservation that goes beyond the protection of small, fragmented, isolated or poorly protected natural forest areas^9^. Besides, strict protection of forest areas would not be sufficient, or even possible, to conserve biodiversity within the global context of land use changes and the growing demand for agricultural products^10,11^.

The agricultural matrix must urgently become an integral element of biodiversity-friendly landscapes, i.e. a force in conserving biodiversity and providing vital ecosystem services to local populations, ensuring livelihoods in degraded landscapes^12^. This is one of the strategic objectives of the Convention on Biological Diversity (https://www.cbd.int/), which specifically recommends going beyond the mere protection of natural ecosystems, and the ecological rehabilitation of those that are degraded. A necessary and complementary alternative is to include biodiversity conservation as a goal in managed ecosystems, and the implementation of biodiversity conservation units within agricultural-forest mosaic landscapes^4^. This is coherent with the theory and practice of Forest and Landscape Restoration, which may enable recovering diversity and ecological functionality within degraded and deforested tropical landscapes ^13,14^.

The need to manage and increase terrestrial biodiversity in agricultural land uses and landscapes in line with the objective of reducing worldwide biodiversity decline and threats to forests and to protected areas, is already recognized ^15,16^. The potential for biodiversity conservation, connectivity and provision of ecosystem services in tropical agricultural landscapes has been the subject of various studies^16–18^. Emphasis is placed on the conservation of tree biodiversity in these agricultural systems, and more specifically on native tree diversity^19^. Indeed, trees in an agricultural matrix represent an effective option for increasing biodiversity in agricultural landscapes. This implies management practices that involve a diversification of trees on farms, for instance in agroforestry and silvo-pastoralism. In fact, increasing tree cover in the agricultural landscape helps to conserve and restore biodiversity in general. Tree cover in such landscapes provides a diversity of habitats, food and facilitates the movement of fauna in the landscape^20^. Tree cover in such landscapes may also improve agricultural productivity^21^. Overall, tree cover is associated with multiple regulating services, including nutrient retention^22^, erosion control^23^, carbon sequestration^24^, pollination^25^, pest and weed control^26^. Moreover, tree products such as timber, firewood and fruit may provide farmers with significant additional income^27^. Besides agro-silvo-pastoral systems, different studies have also highlighted the importance of forest fragments and scattered trees for biodiversity conservation within agricultural landscapes^4,12,15,28,29^.

Within this context, our study was set up as part of the “Trees on Farms for Biodiversity” project (ToNF: https://treesonfarmsforbiodiversity.com/). The project sought to improve biodiversity conservation in agricultural landscapes. This was achieved by building knowledge about the importance of trees on farms from an ecological, social and economic perspective. The project aimed to help producers to improve their livelihoods while reducing their financial and environmental vulnerability.

The aim of the study was therefore to share knowledge on the potential for biodiversity conservation of forests and trees on farms in a human-pressured landscape, which has been little studied in the literature. The study was set up in Honduras, a Central American country with an area of over 11 million hectares, more than half considered forest (6 million hectares)^3^. In the last ten years, Honduras has lost 21,000 hectares of forest annually and its forest landscape integrity index, which measures the degree of modification of the forest due to human pressure, is one of the worst in the world. The intensification of livestock systems and shifting cultivation are considered the main causes of forest degradation and deforestation in the country. Indeed, Honduras faces a high rate of poverty. In 2019, 39.7% of households lived in extreme poverty^30^, and depended directly on natural resource exploitation. 56.5% of the agricultural production comes from familiar agriculture and represents 76% of employment in rural areas. The agricultural sector is therefore one of the most important source of employment, income, exports in the economy of Honduras. More specifically in the study area, 60% of the population depends on agriculture, livestock, fish and hunting to meet their needs, and around 49% of the household lives in extreme poverty.

In Honduras, the study specifically focused on the landscape of Catacamas, recognized as a landscape highly pressured by the implementation of intensive livestock systems. The Catacamas region is important for biodiversity conservation in the region because it borders on three protected areas that are a priority for biodiversity conservation at the regional level. These protected areas are the Río Plátano Biosphere Reserve, the Patuca National Park and the Tawahka Biosphere Reserve. Because of their connection to the Bosawas Biosphere Reserve in northeastern Nicaragua, the protected areas are part of an extensive regional network of protected forest areas that contribute to biological connectivity across Central America. They are therefore key areas for regional conservation initiatives, such as the Mesoamerican Biological Corridor and the “five great forests” of Mesoamerica (https://www.sica.int/noticias/alianza-cinco-grandes-bosques-de-mesoamerica-iniciativa-ambiental-centroamericana-lanzada-en-la-cop25_1_120718.html).

The aim of the study was to characterize taxonomic tree diversity in different land uses in the Catacamas landscape and to assess the potential for biodiversity conservation. The study landscape is characterized by a mosaic of agricultural systems neighboring different types of forest biomes: humid tropical forests, pine and oak forests, dry tropical forests in the lowland and cloud forest in the highlands^31^. We tested the hypothesis that in pressured landscapes such as this, biodiversity conservation was equally provided by agro-silvo-pastoral systems and forest systems, at plot and at landscape scale. We therefore compared tree diversity in different forest and agricultural land uses within the agricultural–forest mosaic landscape of Catacamas in Honduras.

## Results

### Characterization of tree structure and diversity in the Catacamas landscape

In a study area of 84.24 hectares, 15,096 trees belonging to 375 species from which 95% were native, and 74 families were recorded (Table 3). The gamma diversity (maximum estimated species richness across the landscape) plus standard error was 450 ± 23 (Chao1) and 444 ± 11 (ACE).

Fabaceae represented 19% of the total species reported in the study, followed by Lauraceae with just 5% of the total species. From the 74 recorded families, 30 were represented by a single species and 12 by two species. The species *Guazuma ulmifolia*, *Gliricidia sepium* and *Bursera simaruba* represented 33% of the trees inventoried (1851, 1849 and 1229 trees respectively) and 40% of the species reported were represented by three trees or fewer.

The highest percentage of trees (tree abundance) was recorded in live fences (42%), followed by forest fragments (18%), and the lowest percentage of trees was recorded in agroforestry coffee plantations (3%). The highest species richness was reported in forests fragments (193), followed by riparian forest (158) and live fences (154), while the lowest number of species was observed in agriculture (79) and in agroforestry coffee (65) (Table 1).

**Table 1:** Number of species found by land use in Catacamas.

Land uses	Species	Individuals	Singleton species	Doubleton species	Tripleton species	Plot area	Total area (ha)
LF	154	6285 (42%)	29	22	15	0.02	7.24
RF	158	2517 (17%)	35	17	16	0.25	10.5
SFF	193	2778 (18%)	53	20	7	0.5	12
PAST	94	1301 (9%)	29	13	2	1	28
AGRI	70	414 (3%)	26	16	6	1	17
SF	111	1380 (9%)	34	16	12	0.5	5.5
AFC	65	421 (3%)	24	9	4	1	4
Total	375	15,096	81	42	28		84.24

The different land uses are secondary forest (SF), secondary forest fragment (SFF), agroforestry coffee (AFC), agriculture (AGRI) and pastures (PAST), live fences (LF) and riparian forest (RF).

Among the total species in Catacamas, 40% were represented by three or fewer individuals, denoting a high rarity at the level of species in the landscape. Species recorded only once in the study (singletons, represented by only one individual) represented 22% of the total species (81 species). The land use with the highest percentage of rare species was agriculture (68%), followed by agroforestry coffee (57%) and continuous secondary forest (56%). Three land uses showed the highest percentages of species represented by only one individual: agricultural land (37%), agroforestry coffee (37%) and pasture (31%). Live fences, on the other hand, showed the smallest percentage of singletons (18%).

**Figure 1:**
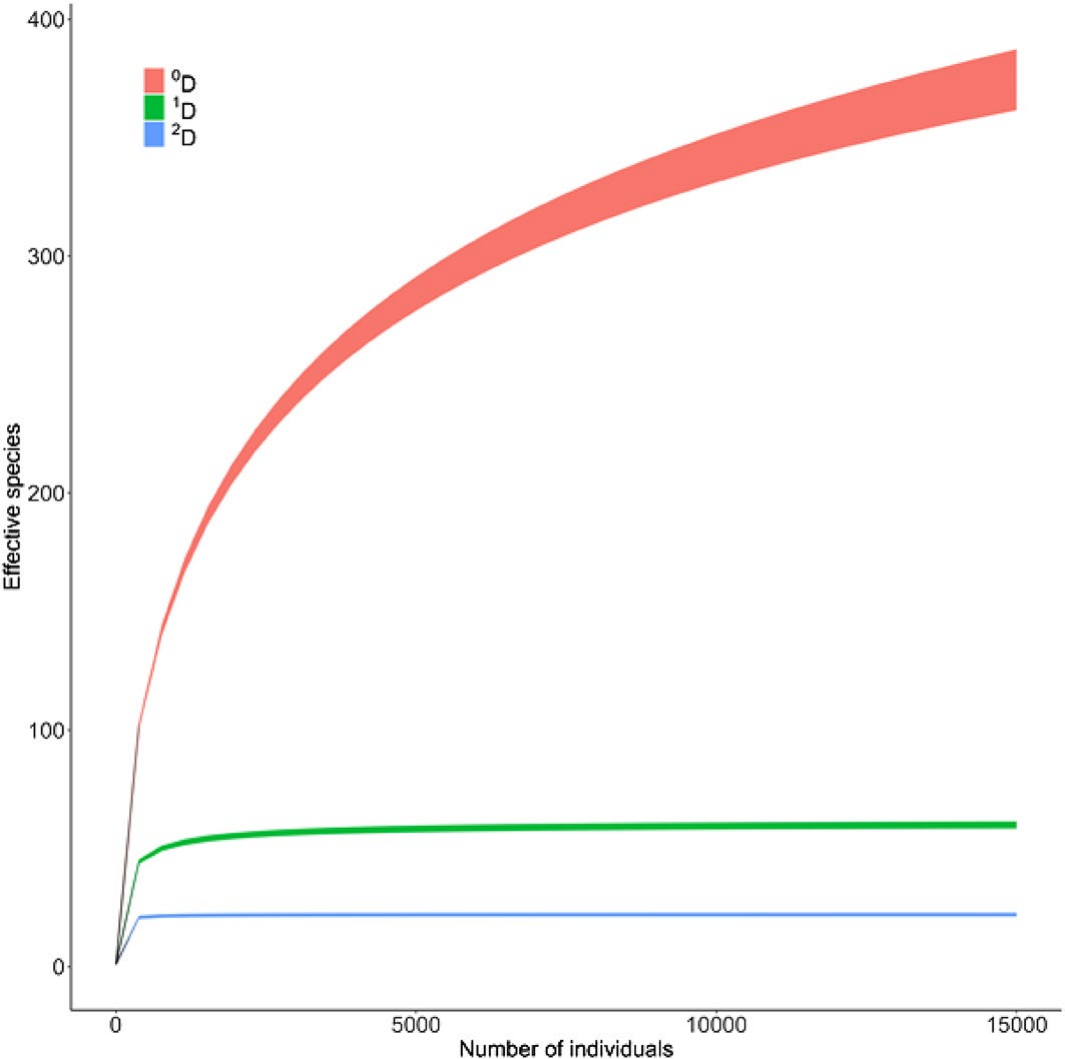
Accumulation curve of effective species for Hill numbers. The curve is based on the Hill numbers of order ^0^D (total effective number of species), ^1^D (effective number of common species) and ^2^D (effective number of dominant species). Colored areas represent 95% confidence intervals.

The accumulation curve for the whole tree community (^0^D, with 375 effective species), indicates a high diversity of species with low abundances. Therefore, registration of many trees is required to reach the maximum observed effective species richness (Fig. 1). The number of effective common species (^1^D, 60 species) peaks with less than 15% of the recorded trees. The same behavior occurs with the dominant effective species (^2^D, 22 species).

#### Distribution of species abundance by land use

**Figure 2:**
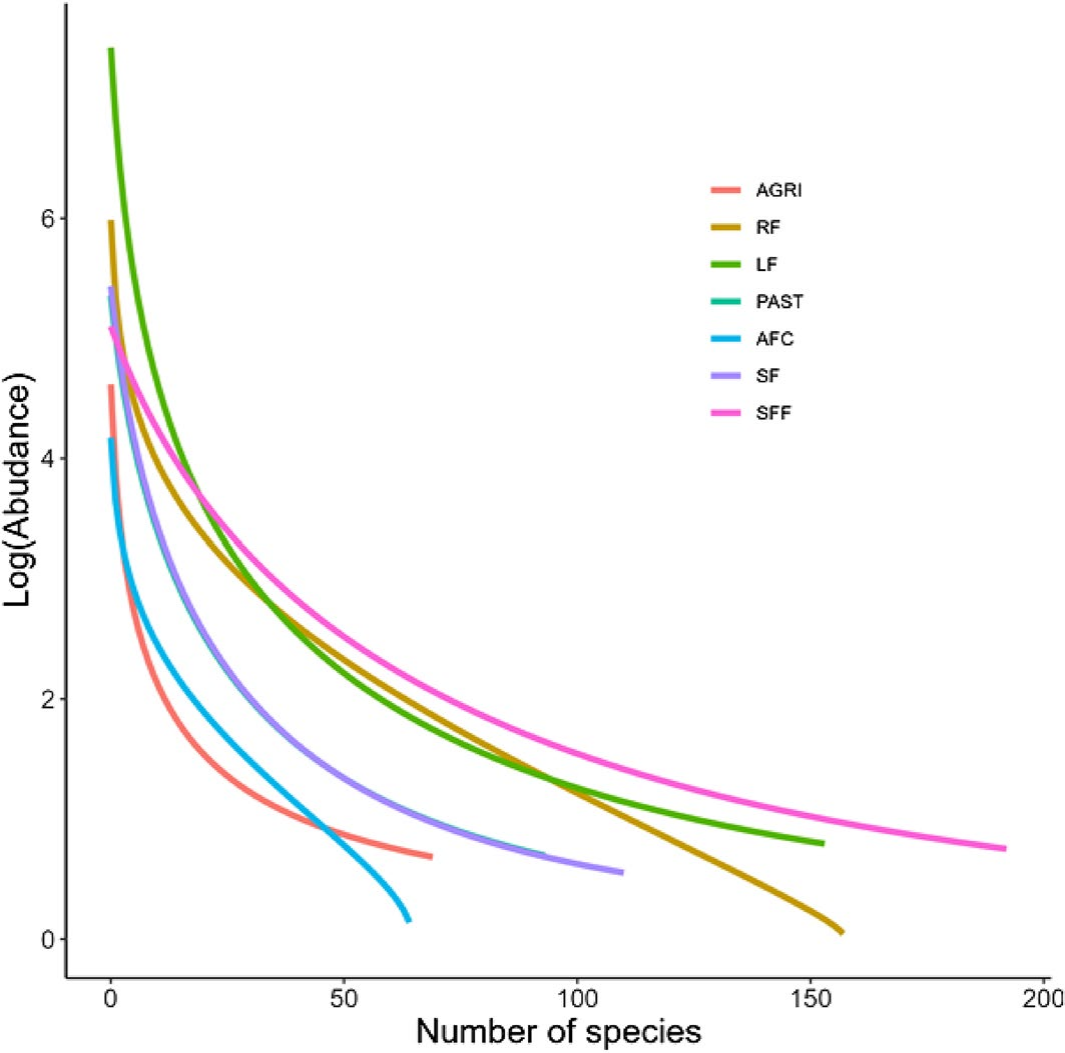
Species rank-abundance curves for each land use. The curves for the secondary forest (SF), secondary forest fragments (SFF), live fences (LF) and pasture (PAST) fit the Zipf-Mandelbrot model. The curves for riparian forest (RF) and agroforestry coffee (AFC) fit the Lognormal model, while the curve for agriculture (AGRI) fits the Zipf model.

Forests fragments, riparian forests and living fences presented the greatest range of species (according to the length of their rank-abundance curves, Fig. 2) and the greatest abundance of species. They also showed a high level of dominance by a few species (indicated by the abrupt decline of curves near the Y-axis, Fig. 2). By contrast, the abundance of species in agriculture and agroforestry coffee showed a more equitable distribution (there is no abrupt decline). Pastures and forests show an intermediate behavior.

Three species were particularly abundant in Catacamas, especially in agricultural land uses, live fences and pasture: *Gliricidia sepium*, *Guazuma ulmifolia* and *Bursera simaruba* (Table 4). *G. sepium* was the most common in live fences (24.9% of total trees) and in agriculture (21.7%), while *G. ulmifolia* was the most common in pasture (17.8%) and was also found dominant in the natural forests, riparian forest (23.7%) and secondary forest fragments (6.1%). In secondary forest the most common species was *Quercus peduncularis* and in agroforestry coffee plantations, it was *Khaya senegalensis* (Table 2). Fig. S1 in the Supplementary Information lists the 60 most common species in the landscape, and the distribution of their abundance by land use.

**Table 2:** List of the five most abundant species for each land use (species that stand out in the rank-dominance curves).

Species	Ind (%)	Species	Ind (%)	species	Ind (%)
AGRI		LF		PAST	
*Gliricidia sepium*	90 (21.74)	*Gliricidia sepium*	1563 (24.87)	*Guazuma ulmifolia*	232 (17.83)
*Guazuma ulmifolia*	61 (14.73)	*Bursera simaruba*	956 (15.21)	*Gliricidia sepium*	121 (9.3)
*Cecropia peltata*	23 (5.56)	*Guazuma ulmifolia*	781 (12.43)	*Bursera simaruba*	120 (9.22)
*Bursera simaruba*	21 (5.07)	Jatropha curcas*	427 (6.79)	*Mimosa tenuiflora*	90 (6.92)
*Tabebuia rosea*	21 (5.07)	*Mimosa tenuiflora*	419 (6.67)	*Byrsonima crassifolia*	69 (5.3)

AFC		SFF		SF	
Khaya senegalensis*	79 (18.76)	*Guazuma ulmifolia*	169 (6.08)	*Quercus peduncularis*	211 (15.29)
*Ilex tectonica*	24 (5.7)	*Byrsonima crassifolia*	159 (5.72)	*Quercus oleoides*	180 (13.04)
*Dialium guianense*	20 (4.75)	*Curatella americana*	147 (5.29)	*Curatella americana*	108 (7.83)
*Brosimum alicastrum*	18 (4.28)	*Aosa grandis*	123 (4.43)	*Pinus caribaea*	100 (7.25)
*Lonchocarpus hondurensis*	18 (4.28)	*Lonchocarpus hondurensis*	117 (4.21)	*Byrsonima crassifolia*	89 (6.45)

RF					
*Guazuma ulmifolia*	596 (23.68)				
*Spondias mombin*	124 (4.93)				
*Cecropia peltata*	88 (3.5)				
*Inga vera*	82 (3.26)				
*Tabebuia rosea*	76 (3.02)				

From these most abundant species, the two non-native are Jatropha curcas and Khaya senegalensis. The different land uses are secondary forest (SF), secondary forest fragment (SFF), agroforestry coffee (AFC), agriculture (AGRI) and pastures (PAST), live fences (LF) and riparian forest (RF).

#### Distribution by diameter class of species number, tree density and basal area per hectare

**Figure 3:**
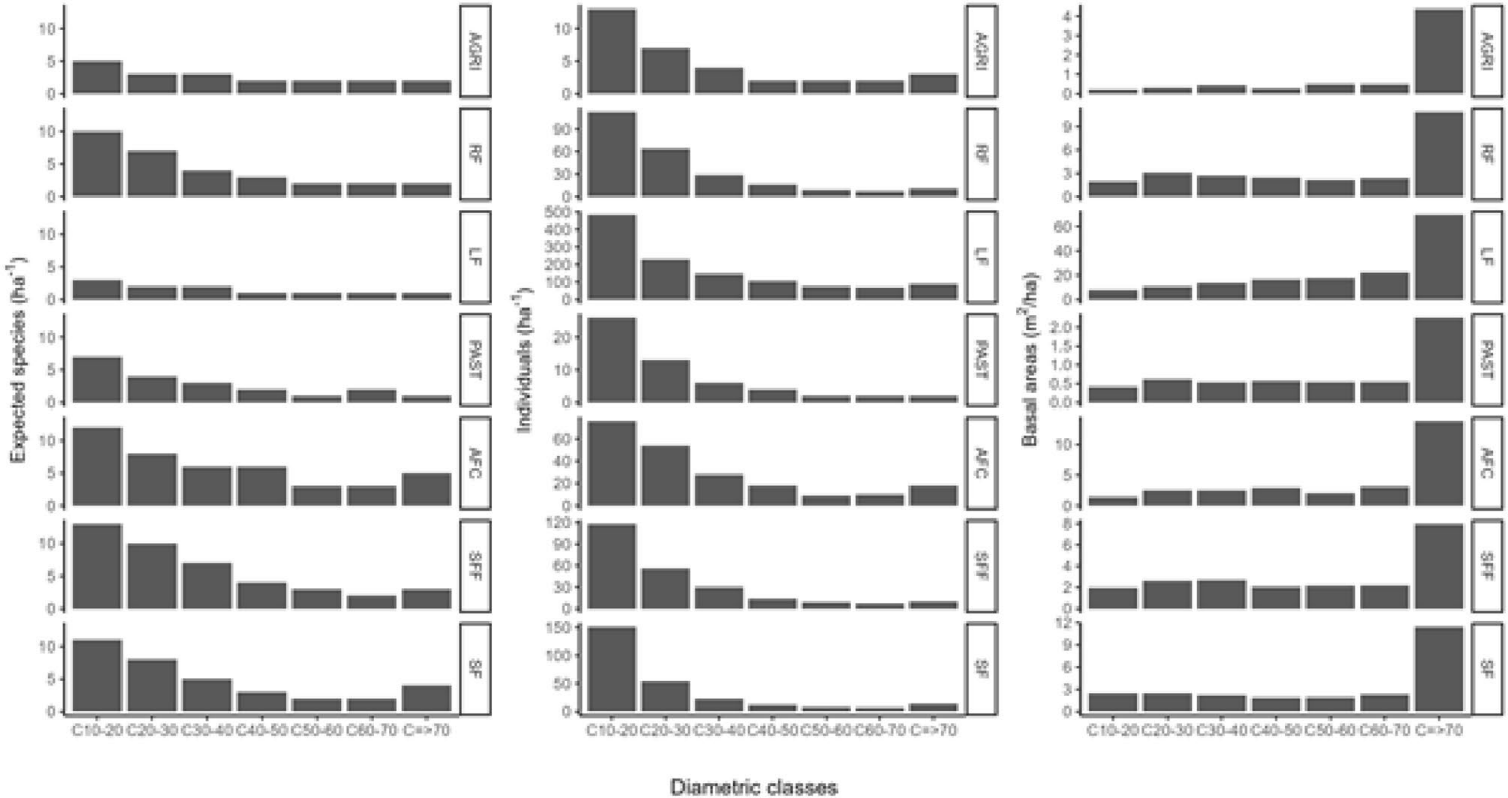
Diameter class distributions for expected species density (first column), tree density (second column) and basal areas (third column) for each land use. The three metrics are standardized per hectare. The different land uses are secondary forest (SF), secondary forest fragment (SFF), agroforestry coffee (AFC), agriculture (AGRI) and pastures (PAST), live fences (LF) and riparian forest (RF).

The greatest species richness and tree density is found in the diameter classes smaller than 30 cm. By contrast, the largest basal areas are in the diameter class greater than 70 cm (Fig. 3). Tree distribution in diametric classes for all the study land uses had an inverted curve shape, indicating high tree densities in smaller diametric classes, and low tree densities in larger diametric classes.

Live fences had the highest tree density and total basal area, but the lowest species richness. Together with agroforestry coffee, live fences stand out from the other agricultural uses because of the high density of trees in lower and higher diametric classes. Agroforestry coffee, secondary forests, secondary forest fragments and riparian forest showed the highest species richness, while PAST and AGRI showed intermediate values. Forests (secondary, secondary fragments and riparian) had higher tree densities than pasture, agriculture and agroforestry coffee systems. The lowest basal area was recorded in pasture and agriculture.

### Diversity in the different land uses

#### Comparison of gamma diversity between land uses

**Figure 4:**
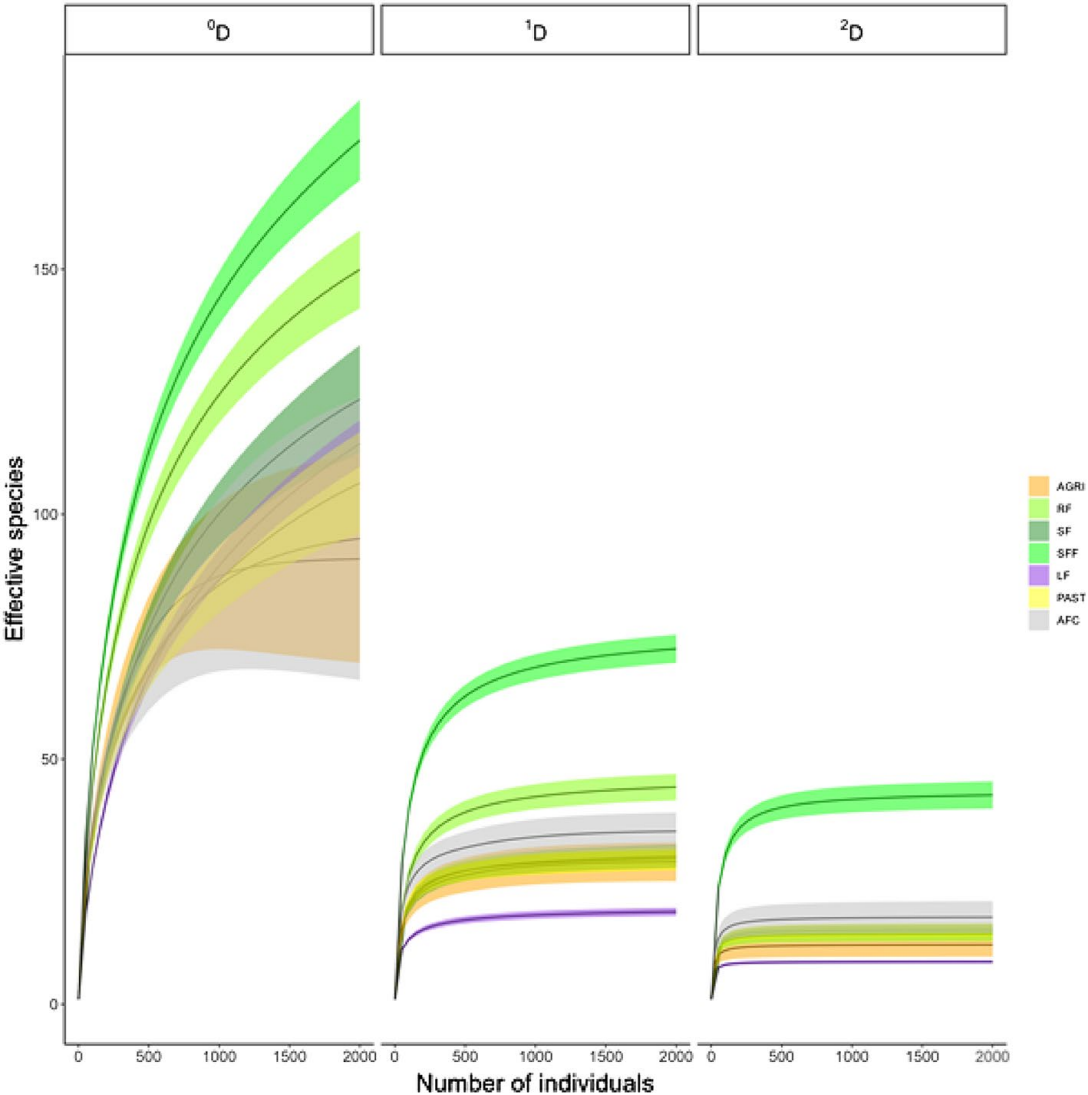
Accumulation curves (interpolation-extrapolation) of effective species for each land use in Catacamas. The curve is based on the Hill numbers of order ^0^D (total effective number of species), ^1^D (effective number of common species) and ^2^D (effective number of dominant species). Colored areas represent a 95% confidence interval.

Secondary forest fragments and riparian forest had the highest number of total effective species (^0^D), followed by secondary forest, while there was no significant difference between the other land uses. (Fig. 4). The highest number of effective common species (^1^D) were found in secondary forest fragments, followed by secondary forest and agroforestry coffee, while live fences showed the lowest number of effective common species. Accounting for dominant species diversity, secondary forest fragments represented the land use with the highest number of effective dominant species (^2^D), while there was no significant difference between the other land uses.

#### Comparison of alpha diversity between land uses

**Figure 5:**
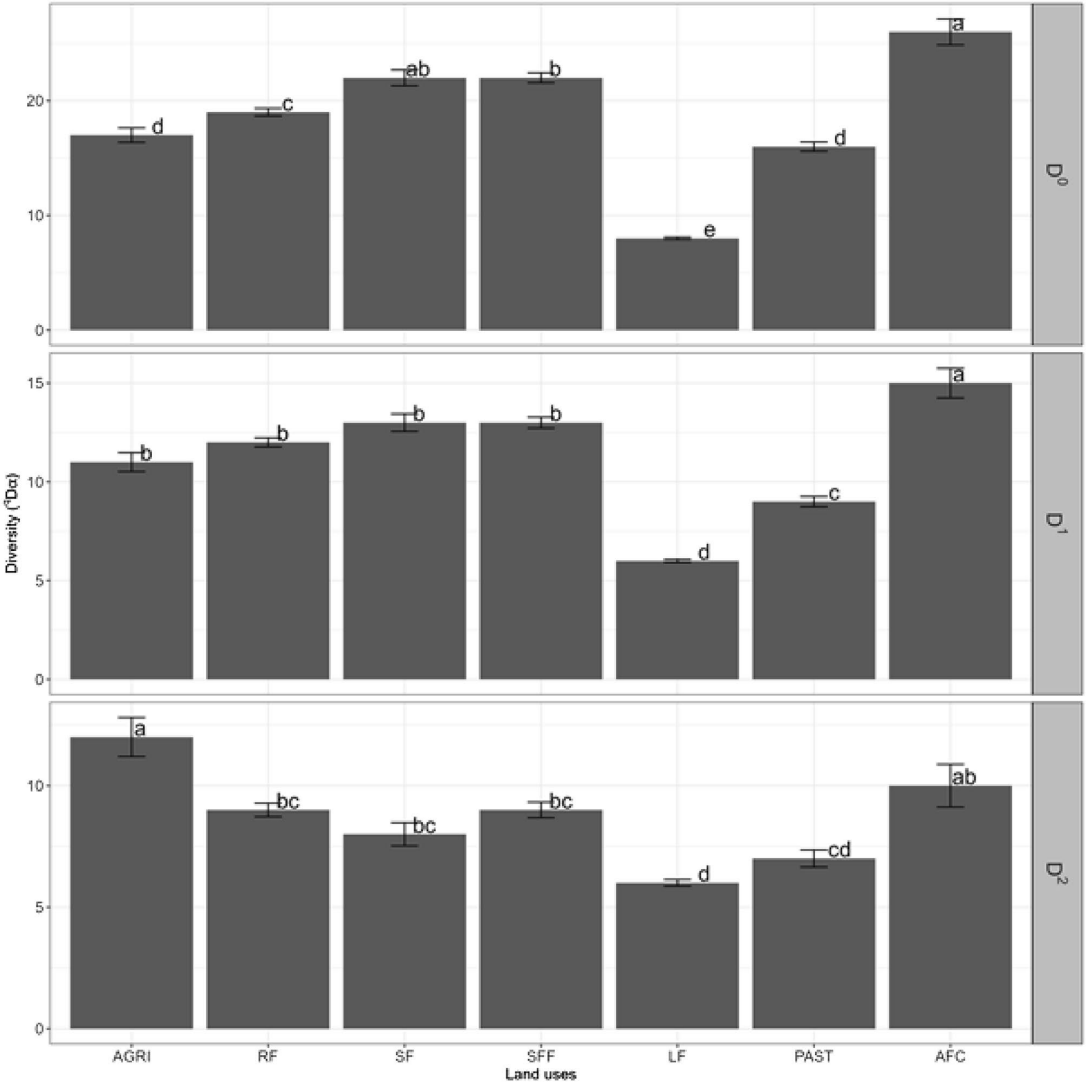
Alpha diversity averages and errors associated with each land use, according to the effective number of species based on Hill numbers of order ^0^D: (total effective number of species), ^1^D (effective number of common species) and ^2^D (effective number of dominant species). The different letters indicate significant differences with α = 0.05.

We found significant differences in average diversity per plot (alpha diversity) in the three estimated Hill numbers. The average effective total number of species (^0^D) was higher in agroforestry coffee, followed by secondary forest fragments. Live fences, pasture and agriculture showed the lowest average values of ^0^D. For the effective average number of common species (^1^D), agroforestry coffee showed greater diversity than the other uses, and live fences and pasture the lowest values. Forests (secondary, secondary fragments and riparian) and agriculture did not differ from each other in the average value of ^1^D. The diversity of dominant species (^2^D) was higher in agriculture than in other land uses. Live fences and pasture presented the lowest diversity value for dominant species (Fig. 5).

#### Comparisons of beta diversity between land uses

**Figure 6:**
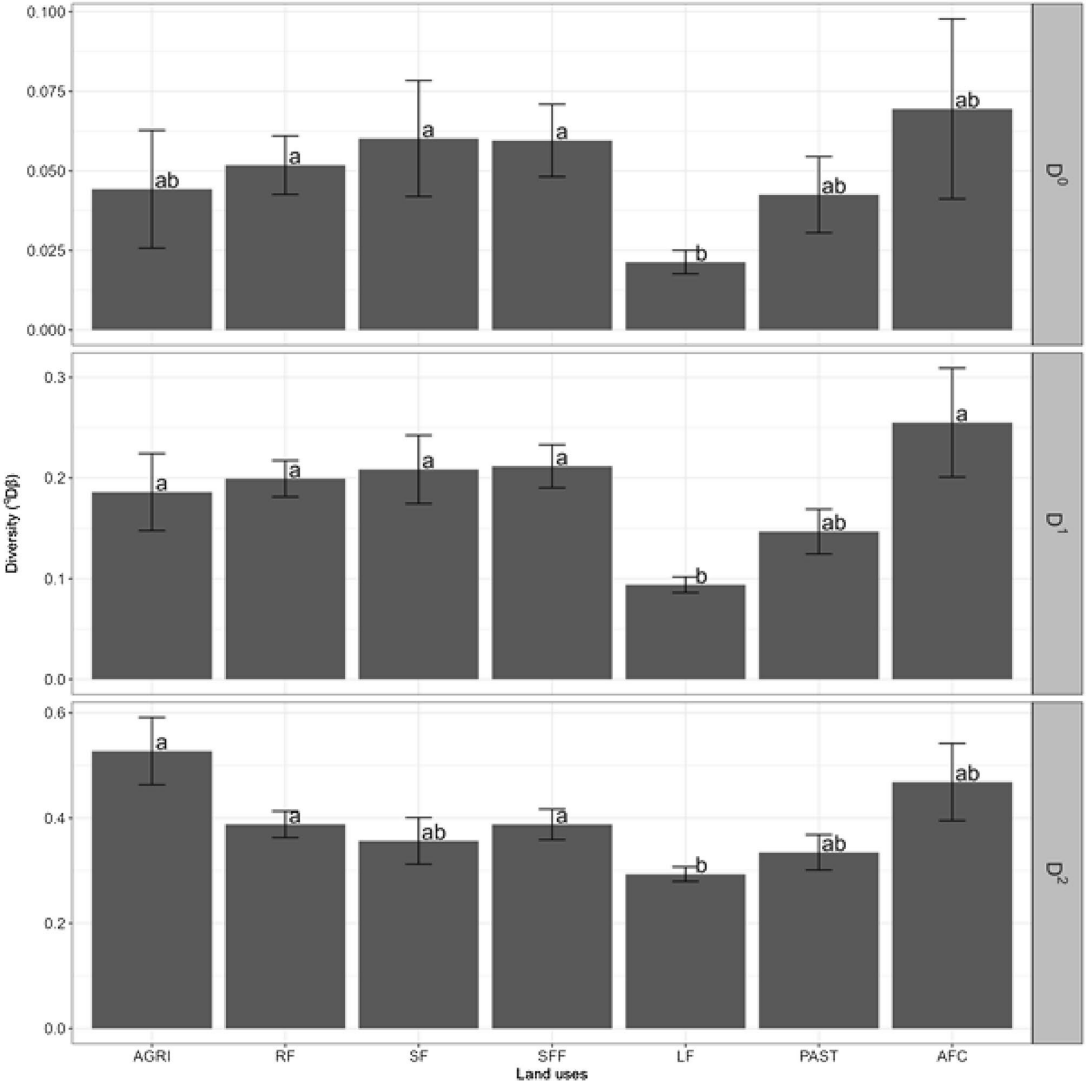
Beta diversity as a specific measure of dissimilarity compared to the total diversity of the landscape. Means and errors associated with each land use, according to the proportion of different species based on the Hill numbers of order ^0^D (total effective number of species), ^1^D (effective number of common species) and ^2^D (effective number of dominant species). The different letters indicate significant differences with α = 0.05.

Beta diversity, as a measure of dissimilarity, presented differences between land uses (Fig. 6). The dissimilarity of the total effective number of species (^0^D) was greater in secondary forest, secondary forest fragments and riparian forests than in live fences. For common species (^1^D), all forests and agroforestry coffee showed greater dissimilarity than live fences. Furthermore, the dissimilarity of the effective number of dominant species (^2^D) was greater in agriculture, secondary forest fragments and riparian forest than in live fences. Forests, agriculture, pasture and agroforestry coffee did not show any difference in the dissimilarity of dominant species.

## Discussion

Although the Catacamas landscape is dominated by extensive livestock production systems, our results indicate that the land uses matrix within the landscape displayed a high diversity of tree species (375 species in the 84.2 ha evaluated), which a huge majority is native (95%). The assessed tree species diversity is characterized by many rare species (40% of the species listed registered three individuals or fewer). Tree diversity is also characterized by very few dominant species: 5% of the total number of species accounted for 66% of all inventoried trees. The highest tree density and diversity are found in diameter classes with a diameter at breast height (DBH) of 10 to 20 cm (52% of total records), indicating a high diversity of young individuals.

In the Catacamas landscape, agricultural land uses are important for landscape biodiversity. For some agricultural land uses such as agroforestry coffee plantations, the biodiversity indices computed were equivalent to the natural forest land uses assessed in the landscape. The potential of agroforestry to conserve biodiversity and some ecosystem services without compromising productivity has been hugely recognized in the literature, and this is related to an appropriate shade management of a diversity of tree species^27^. However, tree diversity and structure differ according to the various agricultural land uses examined in the study.

Live fences are structurally the land use that displays the greatest tree density and basal area. Of the total number of trees, 42% can be found in live fences (about 868 individuals ha^-1^). Tree density in live fences is even higher than in natural forests (231–251 trees ha^-1^). The other cropping land uses have a low tree density: agroforestry coffee plantations (3% of the evaluated landscape) display about 16 trees ha^-1^, agriculture (3%) about 4.1 tree ha^-1^, and pastures (9%) about 3.4 trees ha^-1^.

Live fences, agriculture and pasture have a similar tree composition when accounting for the most abundant species. As found in another studies, these agricultural land uses present an effective total number of tree species (^0^D) that is lower than in the natural forests within the same landscape. All these agricultural land uses differ from forests because the density of trees is concentrated in very few species. This is particularly notable in live fences, where 66% of individuals belong to just five species. These dominant species are heliophytes, fast growing and common in the agricultural landscapes of the region, mainly *Gliricidia sepium*, *Bursera simaruba*, *Guazuma ulmifolia*, *Jatropha curcas*, *Cecropia peltata*, *Tabebuia rosea*, *Byrsonima crassifolia* and *Mimosa tenuiflora*^32^. Actually, these tree species are planted by farmers in living fences, mostly as stakes^33^. In our study area, 61% of the inventoried trees in living fences were established from stakes, 35% were recruited from natural regeneration, and the remaining 4% came from nurseries.

The specific abundance of multipurpose species (e.g. for firewood, fodder and poles) is the result of tree management on agricultural farms in Catacamas^34^. Excluding *Cecropia peltata* and *Byrsonima crassifolia,* the tree species in agricultural uses are frequently planted by farmers. These trees are selected for their tolerance to dry climate and degraded soils, usefulness for shade management (high regrowth and survival following successive pruning) and production of fodder for livestock^32^. *Guazuma ulmifolia* is the dominant species in Catacamas. It is one of the five most abundant species. In our study landscapes, it is found in almost all land uses, except agroforestry coffee and riparian forest. It is an abundant tree species in many secondary forests and pastures in the livestock landscapes of the Central America dry zone. Its characteristics include fast regrowth and early fruiting, at 6–10 years of age. Its seeds have a great capacity to colonize degraded livestock land that is unfavorable for many tree species and its fruit is consumed by livestock, which becomes an effective means of seed dispersal. *Guazuma ulmifolia*, like other species managed by farmers in the region, is therefore highly valued for the ecosystem services it provides: timber, shade and fodder for livestock. This is the reason its regeneration is promoted on livestock farms^35^. Other species such as *Byrsonima crassifolia* and *Tabebuia rosea*, are also found in secondary forests and successfully regenerate in pastures^32^.

Finally, among the agricultural uses in Catacamas, agroforestry coffee plantations stand out for their high species richness, which is greater than tree species richness in other agricultural land uses, and similar to the tree species richness of secondary forest. Agroforestry coffee plantations maintain a high cover of remnant forest trees, compared to other types of agroforestry systems where trees are planted. Therefore its tree species richness is high, as well as the richness of other taxonomic groups that are associated with trees species, such as bats and birds^34^.

Of the five most abundant species in agroforestry coffee plantations in Catacamas, only *L. hondurensis* is recorded among the main species in secondary forest fragments. Almost all the species in agroforestry coffee plantations are native and frequently found in natural forests. Only African mahogany (*K. senegalensis*, Meliaceae), a large multipurpose exotic tree, was introduced in agroforestry coffee. It is of great commercial value for wood, fodder and medicine for livestock and humans^36^. African mahogany was introduced from Africa to Central America in the early 1990s in forest plantations.

Studies in Central America show how certain agricultural practices contribute to conserving and/or restoring biodiversity in agricultural landscapes while improving the well-being of rural families by increasing food production and providing firewood and timber, among other products^37,38^. A number of studies such as the research carried out by Sánchez-Merlos et al.^39^ have found, like our study, significant tree diversity in livestock landscapes in Central America. Increasing tree cover on farms is a key practice in providing complementary habitat, resources and landscape connectivity for a significant group of natural wildlife^34,40^. Silvo-agricultural landscapes function as a buffer zone for the remaining natural areas, contributing to the maintenance of important ecosystem services at the regional level^41^, as is the case with Catacamas landscape examined in our study.

Forest land uses play a key role in the overall diversity of the Catacamas landscape. In this study, continuous and fragmented secondary forest, as well riparian forests, were the land uses that presented the highest effective total number of species (^0^D) and common species (^1^D).

Our results confirm the importance of forest systems for tree diversity conservation in human-pressured tropical landscapes. The value of goods and services provided by secondary and fragmented forests is increasingly recognized^42^. Although not considered equal to mature forests in terms of biodiversity conservation^2^, they may experience a rapid recovery in species richness and associated ecosystem services^42^.

However, the levels of tree diversity found in the natural forests in Catacamas indicate that these systems are degraded or in immature stages of secondary succession. Indeed, age is a key predictor of the contribution of scattered tree species to landscape biodiversity. This variable is however very difficult to assess in tropical modified landscapes^43^. In the present study, we did not have the age range of the study secondary forests following interviews. Future works may consider age estimates through remote sensing analysis of land use history in the region.

Compared to other secondary forests in the region, the forests in Catacamas have very few species per area (per 100 individuals, 23 species in secondary forest, 22 species in secondary fragmented forest and 19 in riparian forest). In remaining tropical dry forests patches in Costa Rica and Nicaragua for example, Gillespie et al.^44^ recorded between 44 to 68 species in 100 m^2^. However, the sites Gillespie et al.^44^ study had different levels of anthropogenic disturbance compared to our sites, as they were located within natural protected or conservation areas. In a broader range, in the neotropics, biodiversity values described by Rozendaal et al.^42^ for secondary forests are, on average, 11 species per 25 individuals with a DBH of at least 10 cm.

In many productive tropical landscapes similar to Catacamas, a high proportion of the forests are fragmented. These fragments tend to be small, isolated and strongly influenced by adjacent agricultural uses, which affects the connectivity and transit of potential seed dispersers. Intensive farming and the presence of livestock can significantly affect natural regeneration in these forest systems^45,46^. In this context, the conservation of tree diversity in surrounding agricultural land uses is crucial and can contribute to strengthening the diversity of forests and landscape in general^20^, especially in landscapes such as Catacamas, under intense human activity and highly fragmented.

## Implications for tree diversity management in Catacamas and in human-pressured landscapes

Our results highlight the potential of tree diversity in agricultural landscape as Catacamas, mainly native tree diversity, and the valuable contribution of agricultural systems. These results are important for a geographically strategic livestock landscape such as this, since it connects and serves as a buffer for surrounding areas of importance for biodiversity conservation in Mesoamerica, such as the Rio Plátano Biosphere Reserve, the Patuca National Park and the Tawahka Biosphere Reserve.

As highlighted in relevant literature regarding the importance of trees in agricultural–forest mosaic landscapes, trees in the different land uses in Catacamas, besides having an intrinsic value as part of the biodiversity of the landscape, may provide a key contribution in terms of fauna habitat and resources, and for the provision of key ecosystem services^4,12,47^. A global meta- analysis confirmed that the local abundance of arthropods, vertebrates and woody plants was 60%–430% higher, and overall species richness was 50%–100% higher in areas with scattered trees than in open areas^29^. Moreover and regarding ecosystem services, the selection and management of trees outside forests in livestock landscapes provide fodder, firewood, fruit and timber. These trees have an ecological, social and economic importance that must be recognized, valued and managed. The present study establishes the knowledge bases for the implementation of biodiversity management actions in the landscape.

The role of agricultural systems in the biodiversity of the Catacamas landscapes is relevant, especially in the case of agroforestry coffee plantations. Despite their small area, they contribute greatly to the diversity of the landscape, particularly because of the total number of species and the number of common species that they contain. Management practices applied to these diversified farming systems, such as the introduction of local tree forest species, may be useful for other less diverse agricultural land uses in the landscape, such as live fences and pasture systems. For land uses such as live fences, agriculture and pastures, which are of low alpha diversity and contribute little to the beta diversity of the landscape, we recommend enrichment practices to increase the number of common species and the total number of species, prioritizing a mix between primary forest species, which are very rare in this landscape, and selected multiple-use species that are useful for farmers.

The application of local producer knowledge, combined with technical knowledge, has proved successful in improving ecosystem services that promote trees, such as agro-silvo-pastoral systems^48^. We recommend meetings with producers of different land uses in order to share experiences the management of trees on farms, tree species use and diversification. The promotion of demonstration sites of good farm management and field schools has shown great value as a methodological approach. This may allow farmers to identify the main problems affecting their production systems and, with their own resources, find biodiversity-friendly solutions that are relevant to their biophysical and socio-economic conditions.

As in many tropical landscapes, the natural forests of Catacamas are secondary and highly degraded forests. This is highlighted by their species composition and low levels of diversity. It may be useful to increase their ecological resilience and economic value by applying active restoration^45,49^. This may consist in further increasing the amount of scattered tree species in the studied landscape, both in terms of area and better structure and increasing the efficiency of ecosystem services they provide. In Catacamas, the restoration of forests could consist of enrichment with primary forest species along with the promotion of regeneration and growth of tree species with multiple uses. Farmers' knowledge of tree species may be useful in this kind of active restoration plan.

## Material and methods

### Study area

**Figure 7:**
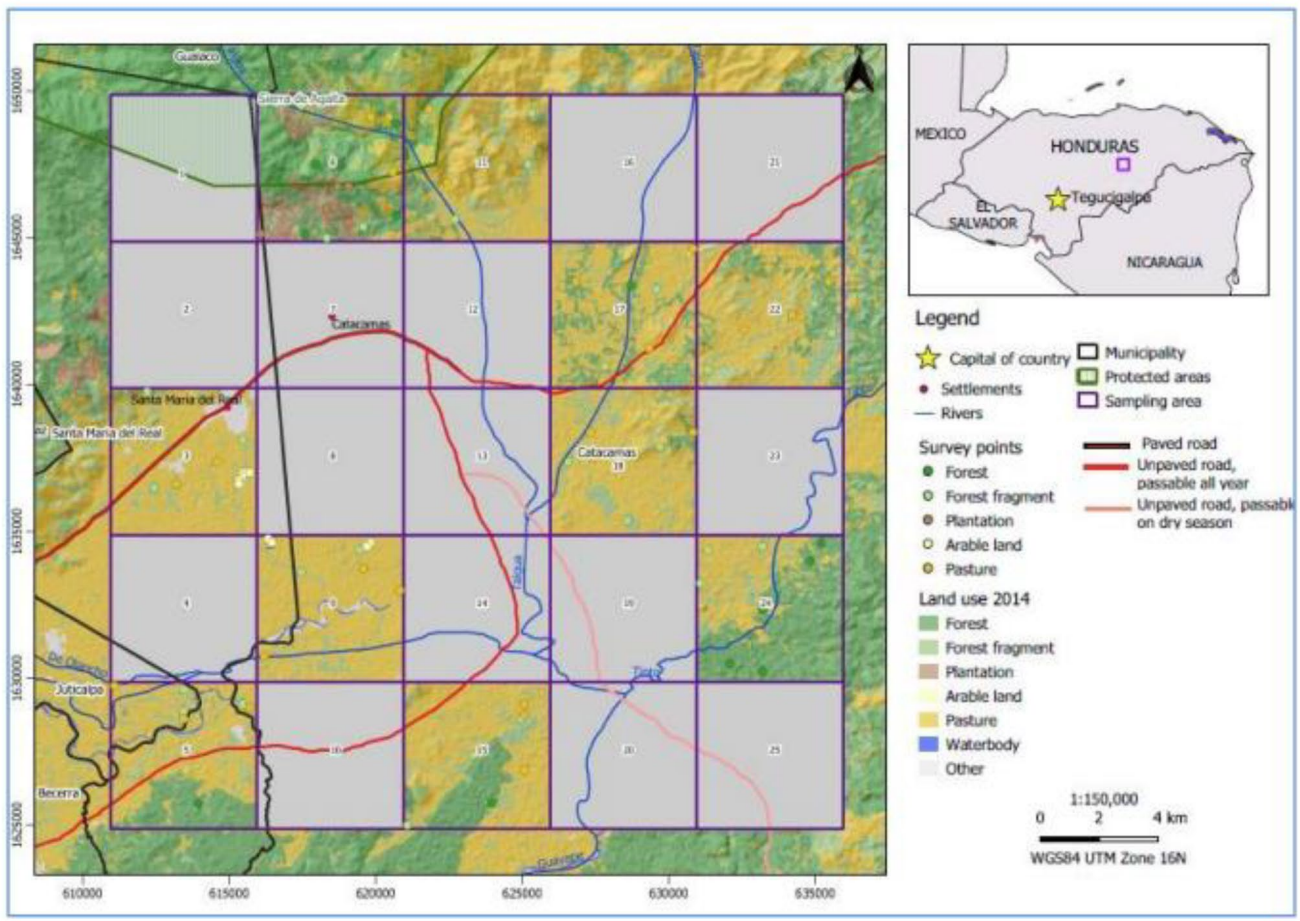
Catacamas landscape (25 km × 25 km) with ten 5 × 5 km sampling quadrats. The map was created using QGIS open source-software version 3.14 (https://www.qgis.org/en/site/). Land use datasets were elaborated by Honduras Ministry of Forestry and shared by the project country team".

The study was carried out in the Catacamas sentinel landscape, a pilot site of the TonF project. The Sentinel Landscapes initiative was created in 2012 by the CGIAR Research Program on Forests, Trees and Agroforestry (FTA). The objective of the initiative is to conduct long-term research using standardized methodologies on the temporal and spatial dynamics of land use, trees and forests in selected territories (https://www1.cifor.org/sentinel-landscapes/home.html). The initiative includes eight sites around the world, representing very different biophysical and socioeconomic contexts, and ends its second and final phase in December 2021. The Catacamas sentinel landscape is located in the municipality of Catacamas, department of Olancho, Honduras (Fig. 7). Catacamas has approximately 133,896 inhabitants and is known for its livestock sector, dairy production, meat and basic grains. The livestock sector is the most productive in the municipality, representing a gross value of more than USD 100 million annually. Livestock production systems are extensive, with low productivity, and have a high impact on natural resources, for example local natural forests. Catacamas is one of the municipalities with the highest deforestation rate in the country, most of which is attributed to implementation of livestock systems.

Within the Catacamas sentinel landscape, the study was conducted specifically in the Guayape Valley. It is an area with vegetation characteristic of tropical dry forest and, to a lesser extent, subtropical wet forest. Forests are mainly secondary in Catamas. The Guayape valley is at an average altitude of 450 m above sea level, with an average annual temperature of 24.7 °C and an annual rainfall of 1235 mm. The north of the study area is connected to the Piedra Blanca Mountain, which is part of the Sierra de Agalta National Park. This park is of great importance for the conservation of biodiversity due to its richness in plant and animal species, including felines and quetzales. The park has an area of 518 km^2^ and is part of the Sierra de Sulaco, which reaches a maximum altitude of 2590 m. The vegetation of the Sierra de Sulaco is characterized by forests of Pinus–Quercus between 700 and 1500 m above sea level, and above by broadleaf cloud forest. In this agricultural-forest landscape, rustic agroforestry coffee plantations (coffee plantations established under thinned forest) and basic grain cropping systems are frequent^50^.

### Sampling design

In the study landscape, an area of 25 km × 25 km (625 km^2^) was delineated and represented an agroecological gradient, following the methodology of the sentinel landscape initiative^51^. This area was further divided into quadrats of 5 km × 5 km (25 km^2^). Ten quadrats were selected using a stratified strategy, with the aim to meet the different target lands used in each selected quadrats. We also took into account quadrat accessibility for field data collection. In each quadrats, vegetation sampling plots were located according to different land uses (Fig. 7). The aim was to have at least four different land uses in each quadrat (Table 3). The land uses considered were: (i) secondary forest (SF), defined as secondary natural woody vegetation of more than 100 ha in area; (ii) secondary forest fragment (SFF), corresponding to secondary forest areas of between 1 and 100 ha; (iii); agroforestry coffee plantations (AFC); (iv) agriculture (AGRI), consisting mainly of basic grains (maize, beans) and (v) pastures (PAST). Plots were also located in live fences (LF) and riparian forest (RF) in the ten selected quadrats.

**Table 3:** Number of plots set up per quadrat in each land use.

ID quadrat	AGRI	RF	SF	SFF	LF	PAST	AFC	Number of land uses	Number of plots
3	6	6		2	50	3		5	67
5		6	1	2	29	3		5	41
6	1	1	4	10	3	1	3	7	23
9	4	8		1	51	3	1	6	68
11	1	4		2	28	1		5	36
15		3	1	1	35	5		5	45
17	2	3			62	4		4	71
18		6		2	49	4		4	61
22	1	2		3	24	2		5	32
24	2	3	5	1	31	2		6	44

The different land uses are secondary forest (SF), secondary forest fragment (SFF), agroforestry coffee (AFC), agriculture (AGRI) and pastures (PAST), live fences (LF) and riparian forest (RF).

### Vegetation sampling

Tree sampling was performed within each plot of the various quadrats, according to each land use category (Table 4), following the protocols of Harrison et al^51^. For the agricultural and pasture land uses, a circular plot of 1.0 ha (with a radius of 56.4 m) was set up. For secondary forest and secondary forest fragments, plots of 0.5 ha (radius 39.9 m) were set up because these land uses had high tree densities. For riparian forest, a rectangular plot of 0.25 ha was delineated. Since riparian forest varied in width in the different plots, the dimensions (width and length) varied for each sampling plot, but their area was always kept to 0.25 ha. For live fences, each sampling plot was a segment that was 100 m long and 2 m wide.

**Table 4:** Number of quadrats, plots and trees in each land use. Plot area and total sampled area are displayed.

Land uses	Number of quadrats	Number of plots	Number of trees sampled	Plot area (ha)	Total sample area (ha)
LF	10	362	11,048	0.02	7.24
RF	10	42	2549	0.25	10.5
SFF	9	24	2784	0.5	12
PAST	10	28	2129	1	28
AGRI	7	17	605	1	17
SF	4	11	1383	0.5	5.5
AFC	2	4	415	1	4

The different land uses are secondary forest (SF), secondary forest fragment (SFF), agroforestry coffee plantations (AFC), agriculture (AGRI) and pastures (PAST), live fences (LF) and riparian forest (RF).

In each plot, we monitored all the trees with a minimum diameter at breast height (DBH) of 10 cm. For each tree, we recorded its species and DBH (around 1.3 m from the soil). For multiple-stem trees, the DBH of each stem with at least the minimum 10 cm was recorded. For trees with broken stems, the DBH and height to the breaking point were measured. Verification of botanical identification made in the field was carried out at family level using the Missouri Botanical Gardens platform (https://www.missouribotanicalgarden.org/) and at the species level using the Taxonomic Name Resolution Service (http://tnrs.iplantcollaborative.org/).

### Data analysis

To characterize tree structure and diversity in the Catacamas landscape, we performed an analysis of diameter distribution by abundance class and total basal area. We also analyzed the total diversity of the landscape.

In order to understand the distribution and structure of tree diversity in the Catacamas landscape, we generated distributions per diameter class per hectare for each land use, for species density, tree density and basal area. The expected species density per hectare in each diameter class was estimated by the rarefaction method^52^. We used the average number of trees per hectare for each diameter class as the number of individuals for the rarefaction (sample size) (Supplementary Information, Table S1).

To visualize the distribution of species abundance, we built rank-abundance curves for each of the land uses. These curves are used to visualize the relative abundance, richness and uniformity of species, in order to identify rare and dominant species^53^. In order to produce the curves, we adjusted different models that assume hypotheses for the distribution of resources and therefore the community structure: steeper curves assume that very few species are sharing the resource, while smoother curves assume that the resource is evenly distributed (Supplementary Information, Table S2).

#### Gamma diversity for the landscape

For the entire landscape, we built species accumulation curves based on Hill numbers of order q (^q^D) from individuals using the interpolation and extrapolation method^54^. The maximum number of individuals to extrapolate was 15,000, which corresponded to the total number of individuals inventoried in the study landscape. Hill numbers represent the actual number of species^55^ that compose the community. The curves were created for the order numbers 0 (^0^D), which is interpreted as the effective number of total species (species richness), with a greater weight for rare species. Order 1 (^1^D) is the effective number of common species and counts species in proportion to their abundance. Order 2 (^2^D) is the effective number of dominant species, which discards all species which occur with low frequency and accounts for the most predominant. We also calculated the maximum expected richness in the landscape using abundance-based coverage estimators (ACE) and Chao1^56^.

In order to assess the potential for tree biodiversity conservation of the different land uses within the Catacamas landscape, we made comparisons of class-level diversity (Gamma diversity for the land uses) and plot-level diversity (alpha and beta diversity for the land uses).

#### Gamma diversity for the land uses

To compare the land uses that we evaluated according to their maximum accumulated diversity, we constructed species accumulation curves based on the Hill numbers of order q (^0^D, ^1^D and ^2^D) from individuals using the interpolation and extrapolation method for each land use. The curves were constructed for 2000 trees corresponding to the average of the total number of individuals recorded for the seven land uses evaluated.

#### Alpha diversity for the land uses

We estimated the average diversity per plot (alpha diversity) based on the Hill numbers of order q = 0, q = 1 and q = 2^54^. We estimate the alpha diversity for each plot through the interpolation-extrapolation method using 100 individuals^54^. This allows us to control the variation in abundances between plots. Subsequently, we performed variance analysis between land uses for each diversity descriptor (qDα), adjusting generalized linear models with negative binomial distribution^57^. Where we found differences, a means comparison test was performed.

#### Beta diversity for the land uses

The total diversity of the Catacamas landscape is a product of the diversity of each land use that is part of it. Therefore, in order to quantify the contribution of each land use to the total landscape diversity, we estimated the actual number of species diversity contributing to total diversity for each sampling plot as a measure of beta diversity (^q^Dβ), expressed as 1/(^q^Dγ/^q^Dα) and interpreted as the proportion of total diversity that, on average, a plot does not share with other plots (dissimilarity). The greater the difference (dissimilarity) with respect to total diversity, the greater the contribution each land use made to the total diversity of the landscape. As in the case of alpha diversity, we performed comparisons between land uses for each beta diversity descriptor (qDβ), adjusting generalized linear models with negative binomial distribution^57^. If differences were found, we carried out means comparison tests.

The analyses were performed using R version 4.0.1^58^. Rarefaction analyses were made with the function rarefi of the vegan package^59^, extrapolation and interpolation analyses with the iNEXT package^60^, and glm with the MASS package^61^, using the function glm.nb. that allows adjustment of generalized linear models with negative binomial distribution. All graphics were built using the ggplot2^62^ and gridExtra packages^63^.

All methods were carried out in accordance with relevant guidelines and regulations.

## Supplementary Information

Supplementary Information.

## Data Availability

The datasets generated during and/or analysed during the current study are available from the corresponding author on reasonable request.
