# Supplementary material for: Tree diversity in a tropical agricultural-forest mosaic landscape in Honduras
Source: Sci Rep. 2022 Nov 3;12:18544. doi: 10.1038/s41598-022-21280-7 (PMC9633691; doi:10.1038/s41598-022-21280-7)

Supplementary Information

Table S1. Average number of individuals (N) per diameter class (DC). These values were used as estimates of tree richness per hectare.

DC (cm)	N
10-20	428
20-30	192
30-40	121
40-50	75
50-60	43
60-70	33
>70	55

Table S2. Selection statistics of the different rank-abundance models for each of the land uses. AIC = Akaike Information Criterion, BIC = Bayesian Information Criterion and D = Deviation.

Land Use	Statistics	Broken-stick	Lognormal	Preemption	Zipf	Zipf-Mandelbrot
AGRI	AIC	405.13	244.32	376.73	**217.68**	217.74
	BIC	405.13	248.81	378.98	**222.18**	224.49
	D	202.57	37.76	172.17	11.12	9.18
RF	AIC	2334.69	**788.39**	1947.14	852.63	854.63
	BIC	2334.69	**794.52**	1950.21	858.76	863.82
	D	1771.9	221.6	1382.35	285.84	285.84
SF	AIC	1395.2	495.03	880.07	535.74	**390.78**
	BIC	1395.2	500.45	882.78	541.16	**398.91**
	D	1038.51	134.33	521.37	175.04	28.09
SFF	AIC	1589.62	826.75	1047.87	1224.39	**730.23**
	BIC	1589.62	833.28	1051.13	1230.92	**740.02**
	D	909.17	142.3	365.42	539.94	43.78
LF	AIC	11242.07	1444.38	5604.59	1405.64	**1029.81**
	BIC	11242.07	1450.46	5607.63	1411.72	**1038.92**
	D	10668.73	867.05	5029.26	828.31	450.48
PAST	AIC	1040.04	368.17	646.57	443.95	**344.08**
	BIC	1040.04	373.26	649.11	449.03	**351.71**
	D	723.63	47.76	328.16	123.54	21.67
AFC	AIC	290.5	**230.77**	275.82	251.66	245.16
	BIC	290.5	**235.12**	278	256	251.68
	D	90.76	27.03	74.08	47.91	39.42

Figure S1. Common tree species of the Catacamas landscape and their abundance by land use.

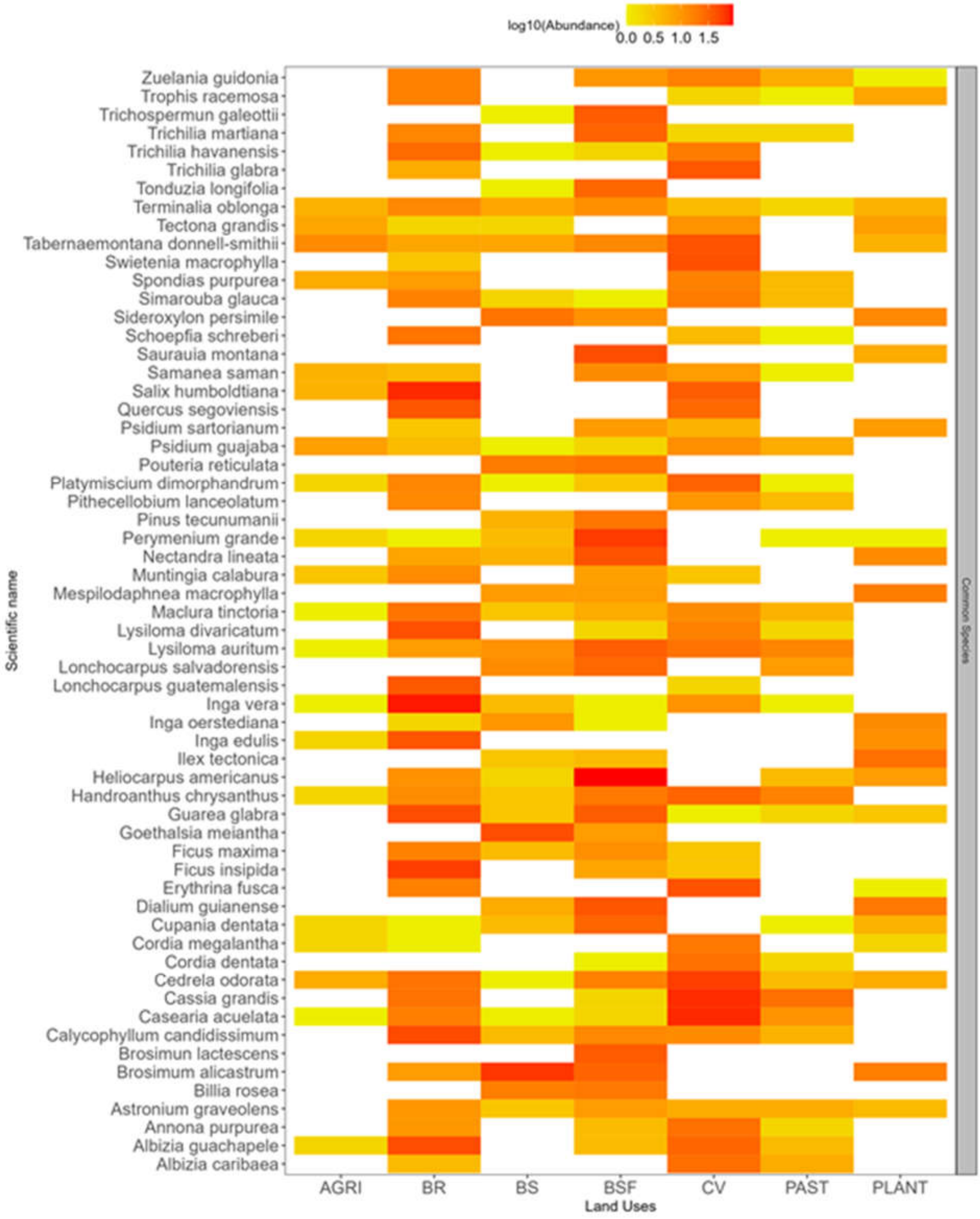

Supplement: Supplementary file 1 — Supplementary Information. [file 41598_2022_21280_MOESM1_ESM.pdf]
